# Location- and Object-Based Representational Mechanisms Account for Bilateral Field Advantage in Multiple-Object Tracking

**DOI:** 10.1523/ENEURO.0519-23.2024

**Published:** 2024-03-20

**Authors:** Christian Merkel, Jens-Max Hopf, Mircea Ariel Schoenfeld

**Affiliations:** ^1^Department for Neurology, Otto-von-Guericke University, 39120 Magdeburg, Germany; ^2^Behavioral Neurology, Leibniz-Institute of Neurobiology, 39118 Magdeburg, Germany; ^3^Schmieder-Kliniken, 69117 Heidelberg, Germany

**Keywords:** event-related potentials, hemispheric specialization, multiple-object tracking, visual attention

## Abstract

Keeping track of multiple visually identical and independently moving objects is a remarkable feature of the human visual system. Theoretical accounts for this ability focus on resource-based models that describe parametric decreases of performance with increasing demands during the task (i.e., more relevant items, closer distances, higher speed). Additionally, the presence of two central tracking resources, one within each hemisphere, has been proposed, allowing for an independent maintenance of moving targets within each visual hemifield. Behavioral evidence in favor of such a model shows that human subjects are able to track almost twice as many targets across both hemifields compared with within one hemifield. A number of recent publications argue for two separate and parallel tracking mechanisms during standard object tracking tasks that allow for the maintenance of the relevant information in a location-based and object-based manner. Unique electrophysiological correlates for each of those processes have been identified. The current study shows that these electrophysiological components are differentially present during tracking within either the left or right hemifield. The present results suggest that targets are mostly maintained as an object-based representation during left hemifield tracking, while location-based resources are preferentially engaged during right hemifield tracking. Interestingly, the manner of representation does not seem to have an impact on behavioral performance within the subjects, while the electrophysiological component indicating object-based tracking does correlate with performance between subjects. We propose that hemifield independence during multiple-object tracking may be an indication of the underlying hemispheric bias for parallel location-based and object-based tracking mechanisms.

## Significance Statement

The human visual system is able to keep track of multiple independently moving objects within the visual field over a period of time. It has been suggested that two separate cognitive resources might exist that are assigned to each of the two visual hemifield. In the current paper, we show that these two resources are implemented within two different representational domains—an object-based within the left hemisphere and a location-based within the right hemisphere. This study bridges the controversy between the previous findings of the parallel involvement of object- and location-based attentional mechanisms during tracking and the intriguing reports of hemispheric independence during tracking.

## Introduction

In daily life, our visual system is continuously challenged by the simultaneously incoming information and manages to separate relevant from irrelevant information. This is particularly difficult when presented with dynamically changing visual information such as during tracking multiple relevant independently moving objects among other distracting ones. The multiple-object tracking (MOT) task (Pylyshyn and Storm, 1988) provides a great opportunity to study the involved processes. During this type of experiment, the subjects are required to visually track a certain number of moving items among other, visually identical items. At first glance surprising, participants are able to keep track of at least four individual objects without much effort (Pylyshyn, 1989, 2004; Scholl et al., 2001; Alvarez and Franconeri, 2007).

Several mechanistic explanations were put forward to account for this ability. They mainly focus on the continuous parametric relationship between behavioral tracking ability and properties of the tracking task like number of tracked items (Pylyshyn, 1989; Alvarez and Franconeri, 2007), item speed, or minimal inter-item distance (Shim et al., 2008; Franconeri et al., 2010; Chen et al., 2013). All these suggestions converge on variations of a continuous shared resource model that suggests either a spatial or temporal split of attentional resources across the relevant targets (Alvarez and Franconeri, 2007; Horowitz and Cohen, 2010; Howe et al., 2010; Chen et al., 2013) or a reduction of tracking resources by spatial interference (Shim et al., 2008; Franconeri et al., 2010).

A further intriguing property of the object tracking task is the behavioral advantage of performing the task across hemifields (bilateral) compared with performing within the left or right hemifield only (Alvarez and Cavanagh, 2005). The capacity for the bilateral tracking almost doubles compared with the unilateral task, suggesting at least in part independent tracking mechanisms for each hemisphere (Alvarez and Cavanagh, 2005; Chen et al., 2013; Stormer et al., 2014; Strong and Alvarez, 2020). This advantage is even present when the factor of spatial interference is controlled for (Holcombe and Chen, 2012). Importantly, continuous resource models outlined earlier potentially account for these findings by assuming two distinct but functionally similar tracking resources implemented within each hemisphere. It is important to note that the postulated continuous resource models rely on a close relationship between tracking performance and the maintenance of target representations within or across hemifields (i.e., more targets, less representational acuity based on less resource or more spatial interference).

A very different mechanistic account for MOT was suggested in which the entirety of relevant items during visual tracking is maintained as an integrated object-based representation and thus facilitates tracking performance (Yantis, 1992). Recently, behavioral (Merkel et al., 2014), electrophysiological (Merkel et al., 2014, 2017, 2022), and functional imaging (Merkel et al., 2015) data from our lab provided strong evidence for such a mechanism. In a series of experiments, the subjects tracked four out of eight moving identical items. After the tracking phase, the subjects had to indicate whether a probe consisting of four items identically matched the entire target set of objects or not. These experiments revealed a full-match effect as an object-based advantage for probe sets, which were fully congruent with the target set. For this effect, we also were able to identify a specific electrophysiological marker, an amplitude modulation of the N170 event-related component for the object-based representation. In parallel, we also observed a continuous parametric location-based representation of the single items reflected by an amplitude modulation of the N270 component (Merkel et al., 2014, 2022). These two modulations were accompanied by enhanced γ oscillations during the tracking phase in subjects, whose behavioral data indicated a location-based tracking strategy and a reduction in γ oscillations during the motion phase of subjects that maintained items mostly using an object-based strategy (Merkel et al., 2022).

Interestingly, a functional bias between location-based and object-based processing of visual information toward one of the two hemispheres has been observed in visual perception (Egly et al., 1994; Marsolek, 1995) and attention tasks (Pollmann and Morrillo, 2003; Wilson et al., 2005). Moreover in a few studies investigating the bilateral field effect in object tracking, a slight general advantage for processing information within the right hemifield has been observed (Holcombe et al., 2014; Strong and Alvarez, 2020). The question arises whether the separate hemifield biases for visual representations of locations and objects can potentially account for the bilateral field advantage observed in tracking tasks.

Here we employ electrophysiological recordings in conjunction with a lateralized MOT task along with established markers to show that location- and object-based multiple tracking mechanisms entail a representational hemifield bias. Tracking within the left visual hemifield relies on an object-based representation, while a location-based representation is primarily employed to track multiple objects individually within the right visual hemifield. The integration of these representations underlies the bilateral field advantage in object tracking.

## Materials and Methods

Twenty-six subjects (19 female) with a mean age of 26.15 (SD, 4.65) preformed in the lateralized object tracking task. Prior to the experiment, a written informed consent was obtained, after which the subjects were instructed about the task. The study was approved by the local ethics board (no. 141/20).

### Stimuli

**Figure 1.**
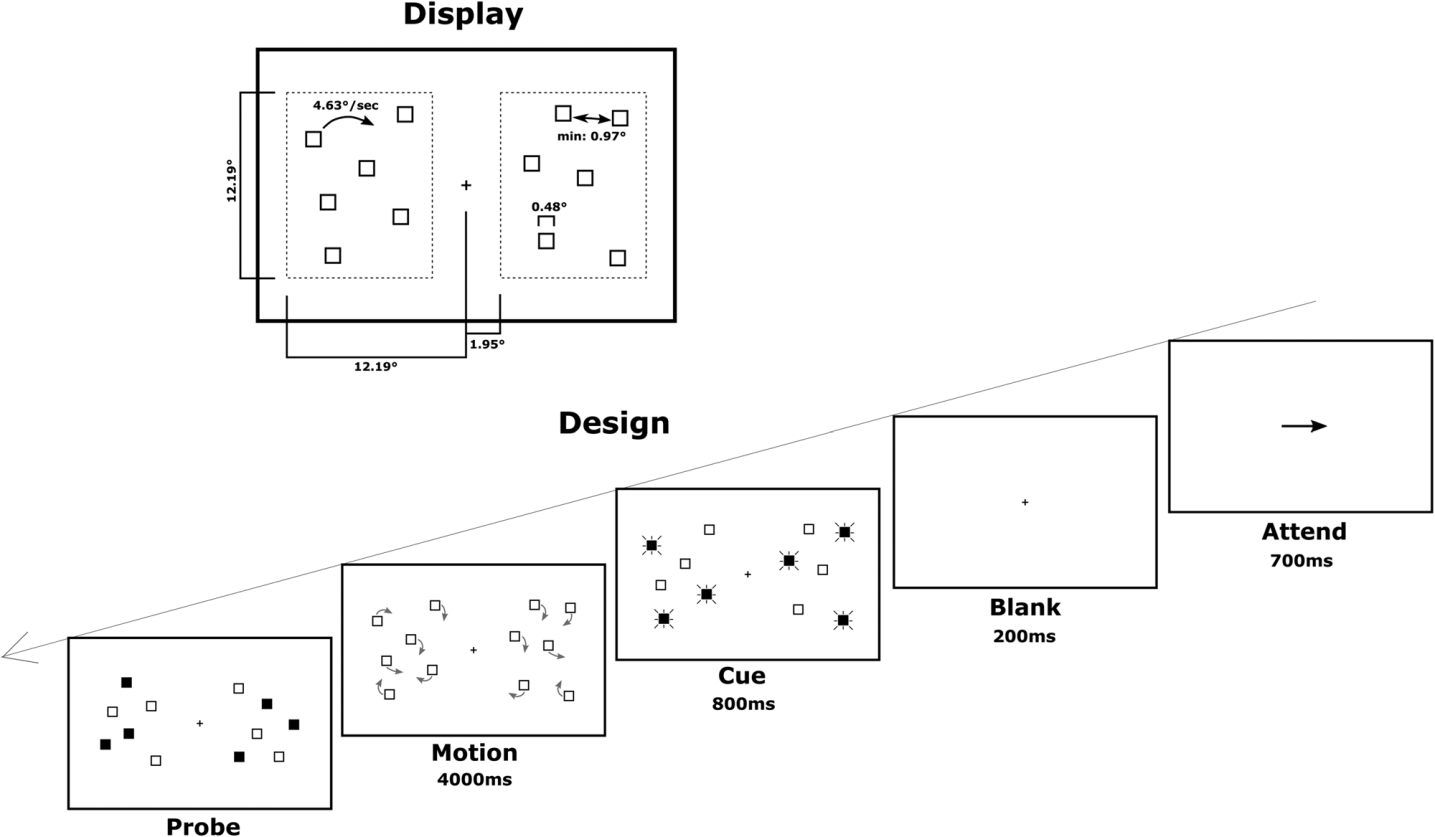
Experimental design. The tracking display contained two sets of six objects each, within the right and left side of the screen, respectively. Each trial started with a directional cue indicating the side of the display the tracking task had to be performed in the current trial. All 12 objects appeared simultaneously on the screen, and three items within the left tracking area as well as three items within the right racking area were cued. Subjects were to attend the cues within the, for the current trial, relevant area only. Subsequently, the two sets of six items moved around their respective areas in a pseudorandom fashion. At the end of the movement phase, three objects of the left and right set of objects each were probed, and the subjects had to indicate whether the probed items within the relevant hemifield fully matched the previously cued items or not.

Throughout the experiment, the subjects were required to fixate a cross of 0.12° located in the middle of the screen. All stimuli were presented in white on a black background. Each trial started with a central directional cue (arrow) for 700 ms instructing the subject to perform the subsequent task either within the left or right hemifield (Fig. 1). After a 200 ms blank period, the outline of 12 squares (0.48°) appeared on the screen. The items were randomly arranged into two sets of six objects allocated within a designated area in the left and right hemifield, respectively. These designated areas occupied a space of 12.19° (vertical) by 10.24° (horizontal) centered at 7.07° left and right of fixation. Thus, the inner vertical border of each area had a distance of 1.95° from fixation. Three of the six items of each set of objects were designated as targets by having them blink twice [changing from an outlined (200 ms) to filled square (200 ms) and back]. At the end of this cueing phase, all 12 items appeared visually indistinguishable from each other in an outlined form again and started to move randomly within their designated (left or right) area with a speed of 4.63°/s for 4 s.

Trajectories for each item and each trial were calculated offline using a semirandomized approach that is described in detail in previous work (Merkel et al., 2014, 2020). The items moved in a continuous and curved manner while not bouncing off the illusory border formed by the respective area or off each other. They kept a minimum distance to any other item of 0.97° throughout the motion phase and never left their area. A unique set of trajectories was calculated for each trial.

Once the motion ceased, again three items of each set of objects appeared as filled squares (probes) at which point the subjects were asked to indicate whether the three probed items within the relevant attended area (left or right) were the identical items designated as targets at the beginning of the trial. Either none of the three probed items on the relevant side were the target items (M0), one of those (M1), two of those (M2), or all three of those (M3, “full match”). The set of probe items appearing within the irrelevant unattended tracking area always consisted of a none-matching probe (M0).

### Procedure

The experiment was performed in a dark shielded chamber, and the subjects were placed 70 cm in front of the display. The subjects were shown up to 10 practice trials to familiarize themselves with the task. Special emphasis was given to the directional cue, and the subjects had to track only the relevant field in each trial. The subjects were instructed to maintain fixation throughout the entire experiment. At the end of each trial, the subjects were to indicate a full match within the relevant tracking area with a button press of the right index finger. Any other partly and nonmatching probes had to be indicated by a button press with the right middle finger. The levels of the two factors “match” (M0/M1/M2/M3) and “hemifield” (left/right) were counterbalanced and presented randomly throughout the experiment. Each of the eight conditions was presented 40 times for a total of 320 trials per subject.

### EEG recordings

While the subjects performed the experiment, the ongoing EEG was continuously recorded using a 32-channel actiCAP system (Brain Products) with electrodes placed according to the international 10–20 system and referenced to the right mastoid. Impedances were kept below 5 kΩ, and the data were acquired with a sampling rate of 500 Hz. Offline analyses were performed using the FieldTrip Toolbox. The recorded data were first offline filtered with a 128 Hz low-pass filter and re-referenced to the average of right and left mastoid. Next, epochs from −400 to 1,000 ms were extracted from the continuous dataset relative to the temporal onset of the probe display. An artifact-reduction step was included by performing an independent component analysis (ICA) decomposition on the epoched datasets using the *runica* function with standard parameters. Components reflecting clear ocular sources were manually selected and consequently rejected. An additional artifact rejection step, removing epochs exceeding individual peak-to-peak thresholds, was performed. Those thresholds were adjusted in order to remove ∼5% of trials within the individual subjects and ranged from 100 to 250 uV.

After extracting epochs reflecting the probe displays at the end of each trial, we additionally focused on the time range of tracking itself. Hereby, epochs were extracted from −400 to 4,000 ms time-locked to the motion onset of each trial. Those epochs were taken from the continuous dataset on which the ICA-based artifact reduction step has been applied to using the components identified during the prior ICA decomposition. In addition, epochs with individualized peak-to-peak amplitudes that included ∼5% of all motion-onset trials per subject were removed.

**Figure 2.**
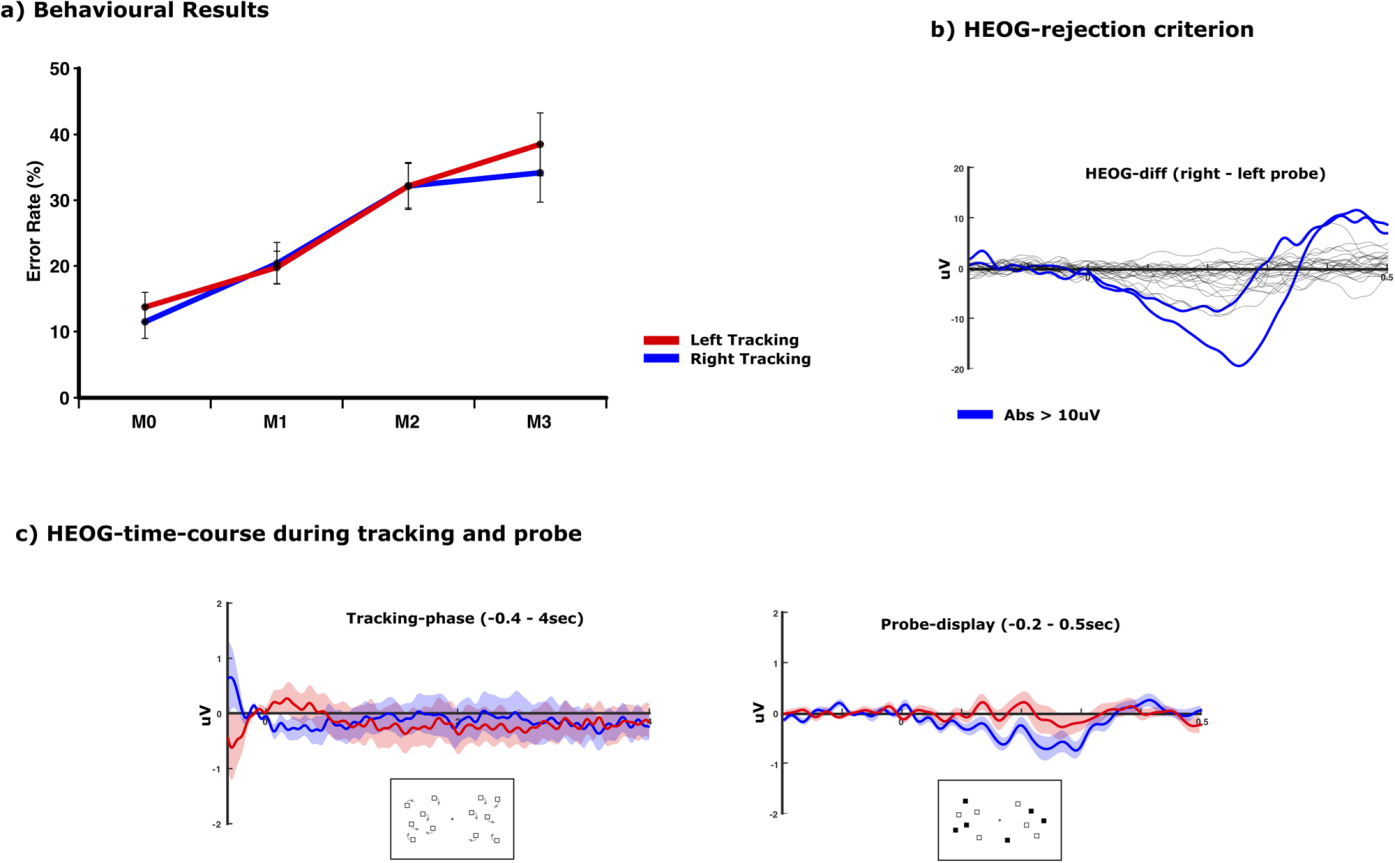
***a***, Behavioral results. Response accuracy decreased for an increasing match between probe items and cued items. This pattern was the same for tracking within the right hemifield and tracking within the left hemifield. ***b***, EOG responses of all 26 subjects toward the appearance of the probe display. All but two subjects (blue traces) exhibited horizontal eye movements of <10 uV. ***c***, Average horizontal eye movements during the tracking phase of the task and the subsequent probe display for tracking within the left and right hemifield (shaded area indicates the standard error). During the probe display, the direction of the horizontal shifts showed a bias toward the relevant tracking area. However, the subjects exhibited sufficient fixation since these shifts were not large enough to modulate the unilateral projection during the lateralized tracking task.

In order to assess the fixation performance of the subjects during the tracking task especially with respect to the experimental factor “hemifield” (left/right), the mean amplitudes of the bipolar HEOG channel (F8–F7 difference) for trials in which tracking had to be performed within the left hemifield versus trials in which tracking had to be performed within the right hemifield were analyzed. This analysis was performed for the probe-display and the motion-phase epochs separately. Figure 2*b* displays the mean left hemifield–right hemifield difference waves within the bipolar HEOG over the time course of the probe display for each subject. Two subjects exceeded amplitudes of 10 uV during this timeframe and were therefore excluded from further analysis. The remaining 24 subjects with mean saccade differences of <10 uV over the course of the probe displays are considered to have been sufficiently fixated throughout the task. Peripheral tracking apertures were placed laterally from 12.19° to 1.95° from fixation. HEOG saccade amplitude estimates typically range from 14 to 20 uV per degree of visual angle (Berg and Scherg, 1991; Picton et al., 2000; Dimigen et al., 2009; Keren et al., 2010). Thus, the range of saccades observed in the subjects can be considered not to have influenced the lateral retinal projection of the separate left and right tracking tasks during the presentation of the probe display. Horizontal eye movements (F8–F7) across the time course of the motion phase and the probe display are displayed in Figure 2*c* for the left hemifield and right hemifield conditions.

The preprocessed data of the 24 included subjects were further baseline corrected from −200 to 0 ms for the probe-display epochs and the motion-onset epochs. Linear trends within all epochs were equally removed. Subsequently, the preprocessed probe-display epochs were averaged over trials for each of the eight conditions [“hemifield” (left/right) × “match” (M0/M1/M2/M3)] separately.

The motion-onset epochs were analyzed by averaging the contra-minus ipsilateral electrode difference waveforms for each trial across the two hemifield conditions (left/right), thereby quantifying the representational load of the tracked items using the contralateral delay activity (CDA) during the motion phase of the tracking trials for left hemifield trials and right hemifield trials (McCollough et al., 2007; Balaban and Luria, 2019).

### Statistical analysis

Subject’s behavioral performance was analyzed as variation in response accuracies toward the different probe displays across the factors “match” and “hemifield” using a two-factorial repeated-measures ANOVA.

During probe-onset epochs, bilateral occipital electrodes exhibited previously described tracking-specific morphologies of N170 and N270 components (Merkel et al., 2014, 2022) in response to the appearance of the probe display. Mean amplitudes were measured for the components within 170–190 ms for the N170 and within 240–300 ms for the N270 across the posterior electrodes P7, P3, Pz, P4, P8, PO9, O1, Oz, O2, and PO10. Repeated-measures ANOVAs were used to analyze mean amplitude variations across the different levels of the factors “match” (M0/M1/M2/M3) and “hemifield” (left/right) for the two components (N170/N270).

The CDA amplitude differences during the motion phase were derived for the left and right hemifield tracking condition at standard occipitoparietal sites P3/4, P7/8, and PO9/10 (Balaban and Luria, 2017) based on the average amplitude between 1,500 and 4,000 ms into the tracking phase.

Next, the subjects were separated into two performance groups based on their relative response accuracies toward the full-match versus the no-match condition [across hemifields (left/right)]. A median split was thus used to categorize subjects as more or less accurate in correctly responding toward the full-match condition. A similar approach to classify subjects by tracking strategy was used previously (Merkel et al., 2015, 2022; Lesch et al., 2020). Subsequently, the ERP variation across factors “match” and “hemifield” of the probe-onset epochs was analyzed for the two groups separately. Additionally, variations of the CDA amplitude during the tracking phase were analyzed with the between-subject factor “group.” Low-performance subjects exhibited a poor accuracy toward the full-match trials, whereas the high-performance subjects showed high accuracies toward the full-matching probes, comparable with the nonmatching probes.

Additionally, hemisphere-specific contributions in amplitude variations toward the N170 and N270 components of the probe-onset epochs were assessed using a three-factorial rANOVA including the factors “hemifield,” “match,” and “electrode sites” [P4, P8, O2, PO10(right) / P3, P7, O1, PO9(left)]. The localization of the N170 component during the left hemifield tracking over left electrode sites and a stronger variation of the N270 amplitudes during right hemifield tracking over right electrode sites motivated an additional analysis considering the differences between left and right electrode amplitudes for the N170 and N270 components. Hemisphere-specific amplitude differences were calculated as [mean(P3, P7, O1, PO9) − mean(P4, P8, O2, PO10)] for left hemifield trials and [mean(P4, P8, O2, PO10) − mean(P3, P7, O1, PO9)] for right hemifield trials. This analysis would exclude any bilaterally evoked amplitude for any of the conditions and only reveal the hemispheric bias for each of the specific patterns (more location-based/object-based) evoked by right and left hemifield tracking, respectively. Interestingly, while the later N270 exhibits a stronger parametric variation in this difference wave for the right hemifield tracking, the N170 modulation during trials in which tracking had to be performed within the left hemifield appears considerably earlier than between 170 and 190 ms within the left electrodes compared with all occipitoparietal sites. The observed modulation of the difference waveform was subsequently analyzed in a time window between 110 and 140 ms.

## Results

The subjects exhibited a parametric variation of performance with higher error rates for trials with more congruity between probes and targets (*F*_(3,69)_ = 12.403; *p* = 0.001; *μ*^2^ = 0.35). Interestingly, the overall pattern in behavior did not differ between tracking within the left and right hemifield (*F*_(3,69)_ = 1.078; *p* = 0.364; *μ*^2^ = 0.045), nor did the mean error rate between left and right tracking (*F*_(1,23)_ = 1.497; *p* = 0.233; *μ*^2^ = 0.061; Fig. 2*a*).

**Figure 3.**
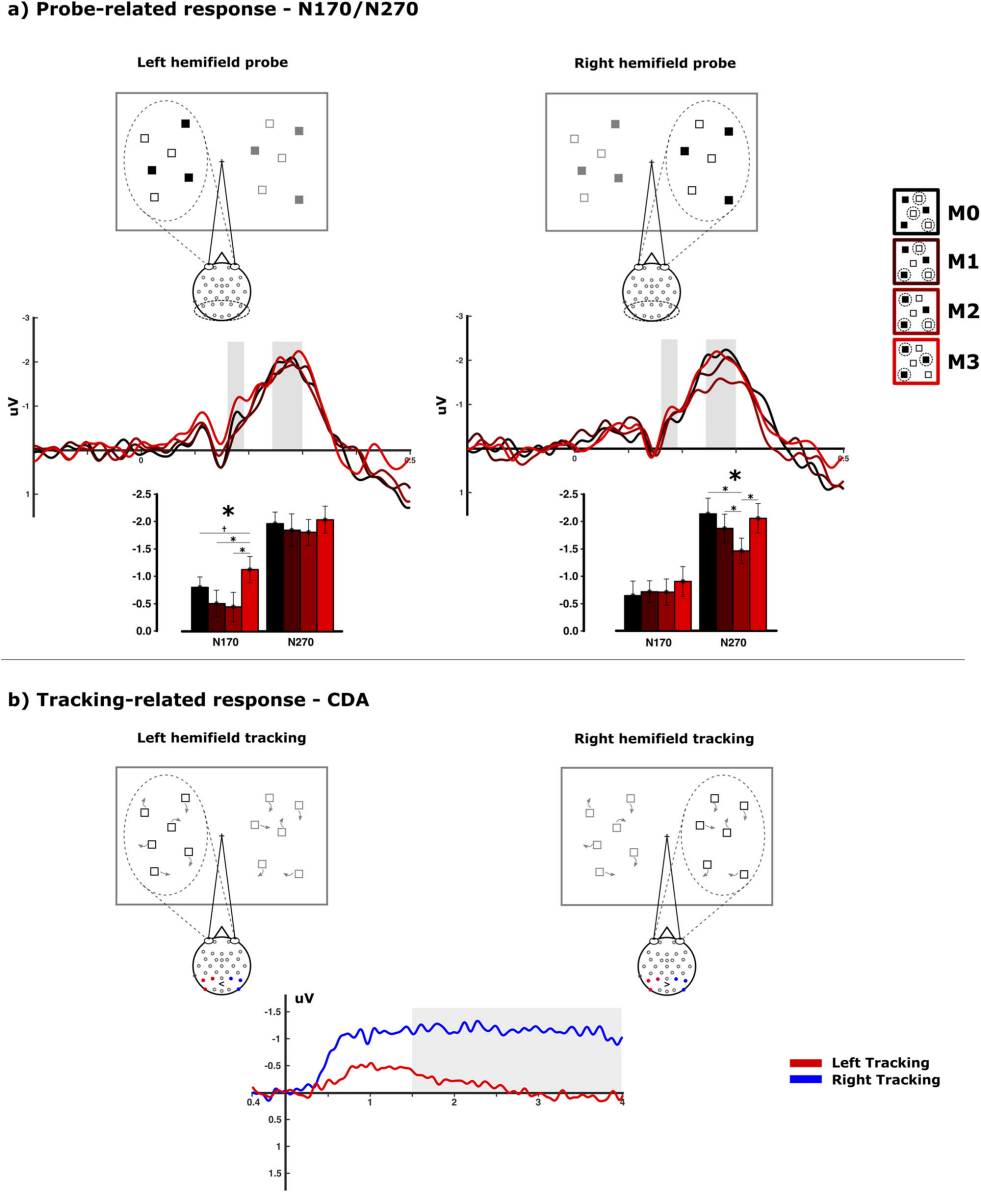
Electrophysiological results [amplitudes in microvolts (uV)]. ***a***, Toward the probe display, the patterns of the N170 and N270 amplitudes within the parieto-occipital electrodes toward the different match conditions (M0–M3) were highly dissimilar when compared with left and right hemifield tracking. The visual probes during tracking within the left side of the screen evoked an exclusive N170 response for the full-match condition. In contrast, tracking within the right side of the screen evoked a more parametric response toward the probe across the different match conditions (significant results are denoted using an asterisk while trends are denoted using a dagger). ***b***, During the tracking phase, a CDA emerged over parieto-occipital electrodes with a larger sustained amplitude for tracking within the right hemifield.

Event-related amplitude modulations for the N170 component were compared using two-factorial rANOVAs including the factors “hemifield” and “match” over the posterior electrodes (Fig. 3*a*). The N170 exhibited a significant amplitude modulation across match conditions (*F*_(3,69)_ = 3.683; *p* = 0.017; *μ*^2^ = 0.138) which did not show an interaction with the attended hemifield (*F*_(3,69)_ = 0.952; *p* = 0.406; *μ*^2^ = 0.040). However, more detailed rANOVAs revealed no significant amplitude modulation for right hemifield trials across the match conditions (*F*_(3,69)_ = 0.438; *p* = 0.709; *μ*^2^ = 0.019), while the main effect for “match” was significant (*F*_(3,69)_ = 3.737; *p* = 0.021; *μ*^2^ = 0.140) when subjects were required to track within the left hemifield. Importantly, this effect for left hemifield tracking trials is driven by an enhanced negativity present exclusively for the full-match condition (M3) compared with that for the partly matching conditions (M1–M3: *t*_(23)_ = 2.648, *p* = 0.014, *μ*^2^ = 0.234; M2–M3: *t*_(23)_ = 2.541, *p* = 0.018, *μ*^2^ = 0.219) with the M0–M3 contrast as a trend (*t*_(23)_ = 1.956; *p* = 0.063; *μ*^2^ = 0.143). An interaction between the hemifield and match conditions with only two levels (M2/M3) remains nonsignificant as well (*F*_(1,23)_ = 1.432; *p* = 0.234; *μ*^2^ = 0.061). However this interaction shows also nonequivalency, meaning that the true interaction effect reaches at least a level of a “smallest effect size of interest” (SESOI). This is the effect size that can be determined to be significant given the power of the test (Lakens, 2014). For the current interaction, the effect is significantly larger than the lower bound of the SESOI (*d* = −0.422: *t*_(23)_ = 3.29, *p* = 0.002) but not lower than the upper bound of the SESOI (*d* = 0.422: *t*_(23)_ = −0.845, *p* = 0.203).

The N270 component did show a significant main effect for the factor “match” as well (*F*_(3,69)_ = 4.660; *p* = 0.007; *μ*^2^ = 0.168), which did not exhibit an interaction with “hemifield” (*F*_(3,69)_ = 1.310; *p* = 0.280; *μ*^2^ = 0.054). Likewise, employing an equivalence test on the 2 × 2 interaction of the N270 component (left/right × M3/M2) does show nonequivalency, suggesting the effect size to be at least relevant with a nonsignificant lower bound (*t*_(23)_ = 0.657; *p* = 0.259) and significant upper bound (*t*_(23)_ = −3.48; *p* = 0.001). Employing one-factorial ANOVAs, a very different pattern emerged compared with the N170 component: the probe display for tracking within the left hemifield did not elicit any variation in mean amplitudes between 240 and 300 ms for the different match conditions (*F*_(3,69)_ = 0.608; *p* = 0.610; *μ*^2^ = 0.026). On the other hand, the probe during tracking within the right hemifield elicited a mostly parametric variation of mean amplitude within the same time range (*F*_(3,69)_ = 5.477; *p* = 0.003; *μ*^2^ = 0.192) with larger negativity for only partly matching probe displays (M1–M2: *t*_(23)_ = −2.178 *p* = 0.040, *μ*^2^ = 0.171; M0–M2: *t*_(23)_ = −3.888 *p* = 0.001, *μ*^2^ = 0.397). No difference was observed for amplitudes elicited by nonmatching and fully matching probes for right hemifield trials within the time range of the N270 component (*t*_(23)_ = −0.458; *p* = 0.652; *μ*^2^ = 0.009).

During the motion phase of the tracking task, a classical sustained CDA modulation was observed (Fig. 3*b*). Most interestingly, this CDA showed a larger amplitude for trials in which tracking had to be performed within the right hemifield compared with trials in which tracking had to be performed within the left hemifield (*t*_(23)_ = 2.139; *p* = 0.043; *μ*^2^ = 0.166).

**Figure 4.**
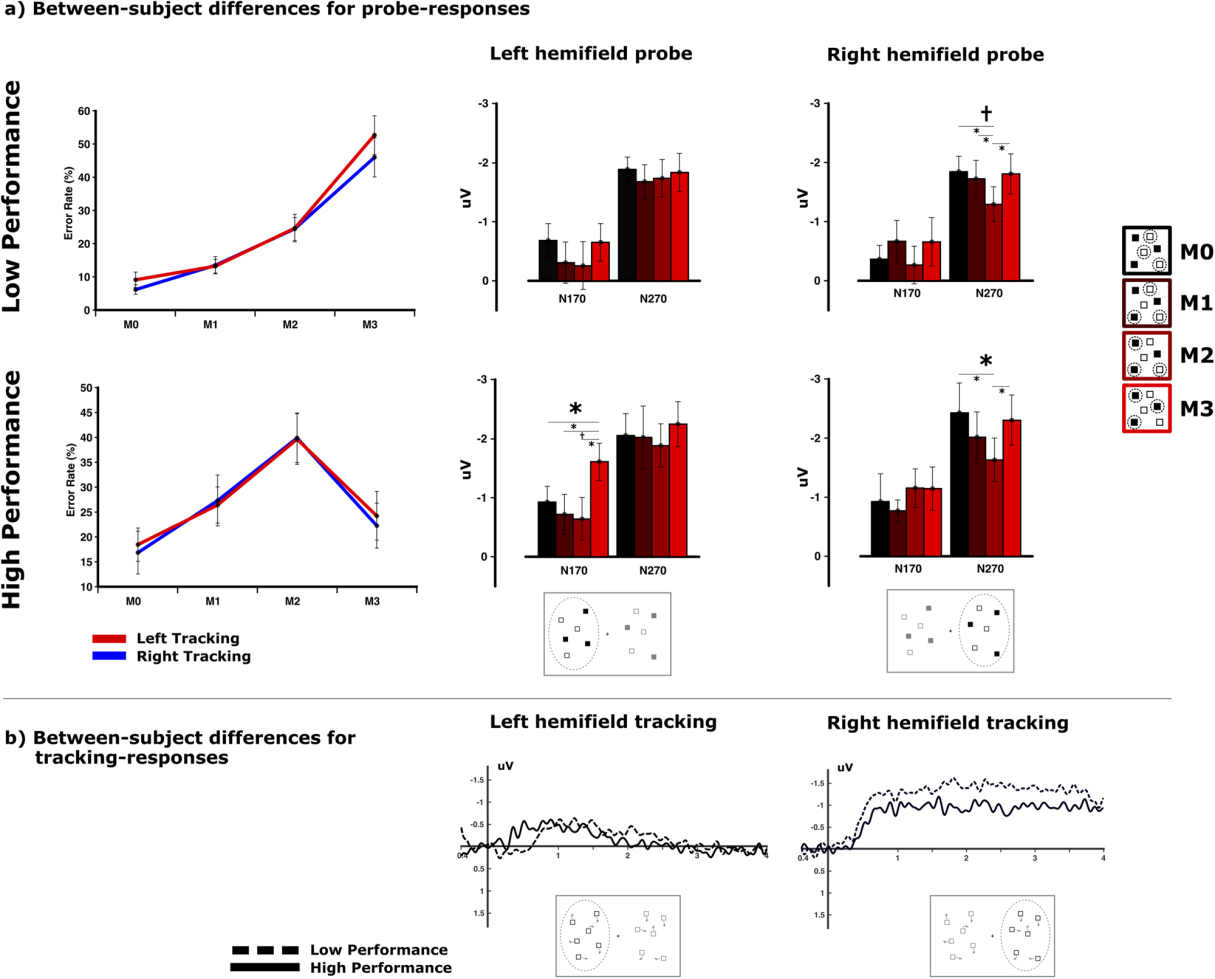
Performance-depending amplitude modulations [amplitudes in microvolts (uV)]. ***a***, During the probe display, the N170 modulation for the left hemifield tracking does show a higher amplitude toward the full match in subjects with better response accuracies in this match condition. The N270 modulation, however, does not show any behavioral correlate between subjects (significant results are denoted using an asterisk while trends are denoted using a dagger). ***b***, No significant interaction can be observed for the CDA amplitude between the left and right hemifield tracking and performance group during the tracking phase.

ERP–amplitude differences toward the probe display across match conditions for left and right hemifield tracking were further analyzed for two different performance groups, which differed in their response patterns toward the match conditions (*F*_(3,66)_ = 17.021; *p* < 0.001; *μ*^2^ = 0.436). This comparison revealed a performance-dependent modulation of the N170 component exclusively for the left hemifield tracking trials (Fig. 4*a*). Hereby, the low-performance group did not show any N170 modulation across match conditions (*F*_(3,33)_ = 1.169; *p* = 0.331; *μ*^2^ = 0.096), whereas the high-performance group did exhibit a significant effect of “match” in the N170 amplitudes during left hemifield tracking (*F*_(3,33)_ = 3.151; *p* = 0.040; *μ*^2^ = 0.223). This effect is driven by a higher negativity for the fully matching compared with that for the partly matching probes (M3–M0: *t*_(11)_ = 2.923, *p* = 0.014, *μ*^2^ = 0.437; M3–M1: *t*_(11)_ = 2.160, *p* = 0.054, *μ*^2^ = 0.298; M3–M2: *t*_(11)_ = 2.378, *p* = 0.037, *μ*^2^ = 0.340). The pattern of N270 amplitudes however does not show any differences between match conditions in either the low- (*F*_(3,33)_ = 0.250; *p* = 0.838; *μ*^2^ = 0.022) or high-performance group (*F*_(3,33)_ = 0.537; *p* = 0.643; *μ*^2^ = 0.047) for left hemifield tracking. A very different picture emerges for tracking within the right hemifield: the N170 does not show any significant match effect for either performance group (low: *F*_(3,33)_ = 0.912, *p* = 0.437, *μ*^2^ = 0.077; high: *F*_(3,33)_ = 0.518, *p* = 0.673, *μ*^2^ = 0.045). However, the N270 exhibits a main effect for “match” in both groups (low: *F*_(3,33)_ = 2.658, *p* = 0.066, *μ*^2^ = 0.195; high: *F*_(3,33)_ = 2.859, *p* = 0.052, *μ*^2^ = 0.206), displaying a similar modulation that appears to be independent of the performance group (*F*_(3,66)_ = 0.270; *p* = 0.832; *μ*^2^ = 0.012).

Most interestingly, within each performance group, the differences in N170 and N270 amplitude patterns between left and right hemifield tracking do not seem to be accompanied with different behavioral patterns for left and right hemifield tracking. Response accuracies across match conditions between trials in which subjects had to track objects on the left side and trials in which subjects had to track objects on the right side are virtually identical within both performance groups (low: *F*_(3,33)_ = 1.070, *p* = 0.368, *μ*^2^ = 0.089; high: *F*_(3,33)_ = 0.182, *p* = 0.884, *μ*^2^ = 0.016).

The CDA amplitude during the motion phase for left and right hemifield tracking did not show any significant interaction with the performance group (*F*_(1,22)_ = 0.085; *p* = 0.773; *μ*^2^ = 0.004; Fig. 4*b*). Likewise, high- and low-performing subjects do not differ in their overall CDA amplitude for left and right hemifield tracking (*F*_(1,22)_ = 2.102; *p* = 0.161; *μ*^2^ = 0.87), although the CDA difference between subjects does visually exhibit a different temporal morphology for left and right hemifield tracking.

**Figure 5.**
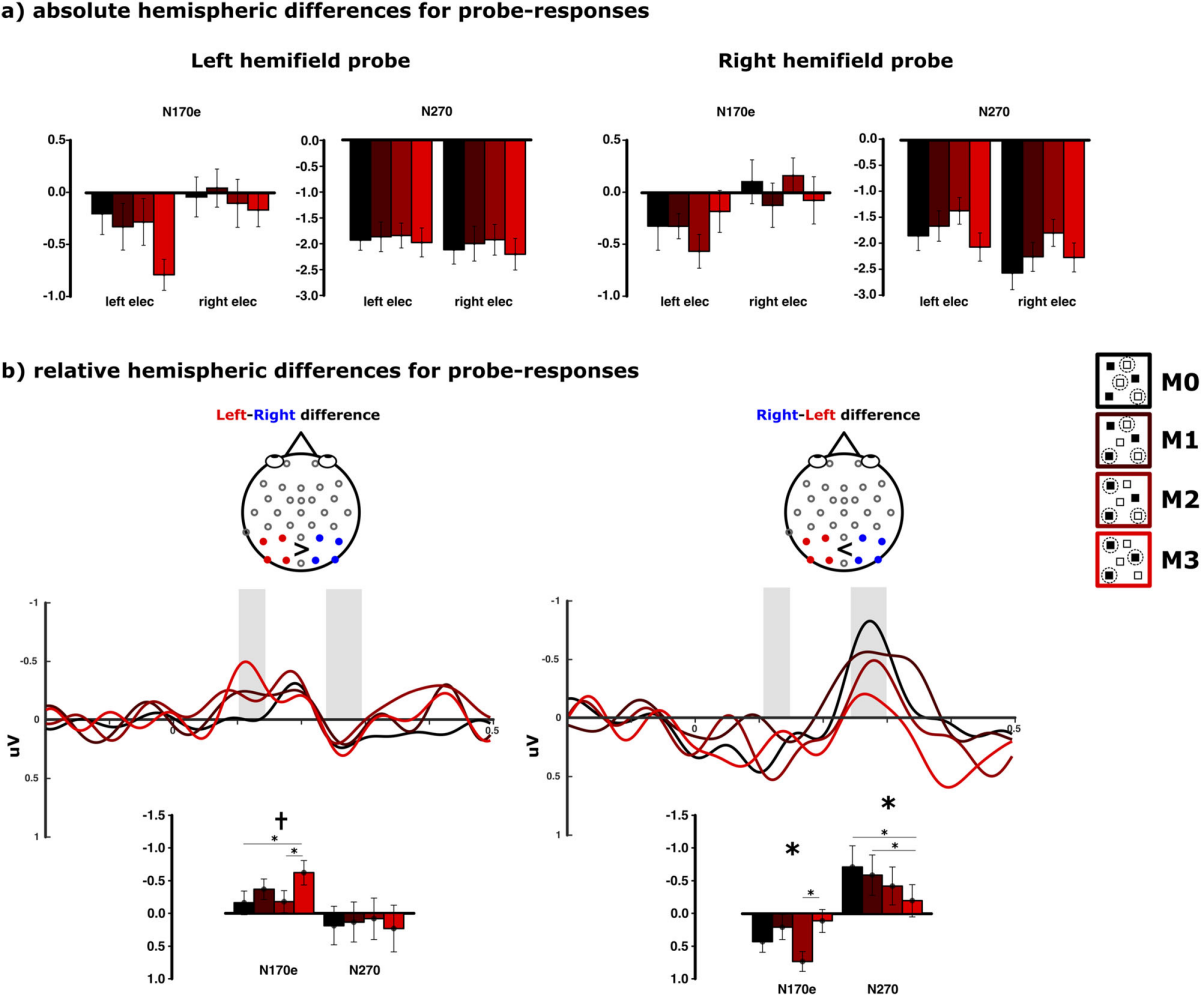
Hemisphere-specific amplitude modulations for the probe responses [amplitudes in microvolts (uV)]. ***a***, During left hemifield trials, the N170 component appears within left electrode sites, whereas during the right hemifield tracking, the N270 modulation has a right hemispheric bias. ***b***, Left–right electrode difference waveforms for left hemifield tracking trials and right–left difference waveforms for right hemifield tracking trials. The N170 appears to originate from the left hemisphere, and the N270 has a right hemispheric bias (significant results are denoted using an asterisk while trends are denoted using a dagger).

Next, we sought to investigate potential hemispheric biases for the N170 and N270 components for left hemifield and right hemifield tracking, respectively. Therefore, we included electrode side [PO9, P3, P7, O1 (left) vs PO10, P4, P8, O2 (right)] as a factor in a three-way repeated-measures ANOVA (“hemifield” × “side” × “match”) for the N170 and N270 amplitudes (Fig. 5*a*). During the early N170 time range, amplitudes were more negative for the left compared with the right occipital electrodes across all trials (*F*_(1,23)_ = 9.741; *p* = 0.005; *μ*^2^ = 0.298). Importantly, this amplitude difference between left and right electrodes was not different for probes presented within the right and left hemifield (*F*_(1,23)_ = 0.097; *p* = 0.758; *μ*^2^ = 0.004). The three-way interaction between “hemifield,” “side,” and “match” condition was significant (*F*_(3,69)_ = 5.399; *p* = 0.003; *μ*^2^ = 0.190) giving an indication together with Figure 5*a* for the specific full-match effect of the N170 during left hemifield trials being more prominent within the left hemisphere. The mean amplitudes of the N270 component did not differ for left and right electrodes across hemifields and match conditions (*F*_(1,23)_ = 1.309; *p* = 0.264; *μ*^2^ = 0.054). The N270 shows however a significant variation for the match conditions across all electrodes and hemifield conditions (*F*_(3,69)_ = 4.849; *p* = 0.006; *μ*^2^ = 0.174). Interestingly, the N270 component appears more negative across match conditions within the right electrodes compared with the left electrodes only for right hemifield trials (*F*_(1,23)_ = 6.034; *p* = 0.022; *μ*^2^ = 0.208).

Given the N170 component exhibiting the match effect during left hemifield tracking mostly within the left hemisphere and the N270 component being more negative for right hemifield tracking within the right hemisphere, N170 and N270 amplitude variations were assessed as a difference wave by subtracting left-minus-right electrode signals for left hemifield trials and right-minus-left electrode signals for right hemifield trials (Fig. 5*b*). Hemisphere-specific N170 and N270 amplitude modulations were assessed using a three-factorial rANOVA across factors “component,” “hemifield,” and “match.” This analysis confirmed a location bias for the N170 within the left hemisphere and the N270 within the right hemisphere, respectively, as a strong trend toward an interaction between “component” and “hemifield” (*F*_(1,23)_ = 3.635; *p* = 0.069; *μ*^2^ = 0.136). The detailed pattern of the right-biased N270 difference wave during right probes across match conditions shows a strong linear variation along all four match conditions (linear contrast: *F*_(1,23)_ = 9.634; *p* = 0.005; *μ*^2^ = 0.295) with a higher amplitude for a greater mismatch between the probe and targets (M0–M3: *t*_(23)_ = −3.067, *p* = 0.005, *μ*^2^ = 0.290; M1–M3: *t*_(23)_ = −2.379 *p* = 0.026 *μ*^2^ = 0.197). Please note that the deviant high amplitude for the full-match condition during this time range, observed across all posterior electrodes, disappears in the difference waveforms. The modulation of the early N170 difference wave for the left hemifield trials across the match conditions just fails to become significant (*F*_(3,69)_ = 2.543; *p* = 0.071; *μ*^2^ = 0.100). During full-match trials, however, the amplitude between 110 and 140 ms of the difference waveform is more negative than that during M0 (*t*_(23)_ = 2.743, *p* = 0.012, *μ*^2^ = 0.246) and M2 (*t*_(23)_ = 2.168, *p* = 0.041, *μ*^2^ = 0.17) trials. For the same time range, an amplitude modulation can be observed for the right hemifield trials (*F*_(3,69)_ = 3.741; *p* = 0.019; *μ*^2^ = 0.140) with a higher negativity for the M2 compared with the M3 condition (*t*_(23)_ = 3.045; *p* = 0.006; *μ*^2^ = 0.287).

## Discussion

The present study investigated potential functional dissociations between location- and object-based representational mechanisms during unilateral MOT within the left or right hemifield. The electrophysiological markers for location-based and object-based tracking strategies established previously (Merkel et al., 2014, 2022) permitted the assessment of the relative contributions of both processes for tracking within the left or right hemifield: while tracking three out of six visually identical objects, the subjects consistently exhibited a sustained small CDA amplitude in combination with an early N170 amplitude modulation for subsequent probes that matched fully the relevant set of target items only when the task was performed within the left hemifield. In contrast, tracking multiple items within the right hemifield leads to an enhanced CDA amplitude throughout the motion phase with a parametric variation of the N270 amplitude modulation for probe displays with a varying degree of target–probe congruities. Most interestingly, the amplitude of the N170 component toward the fully matching probes for the left hemifield tracking task is related to the behavior toward this fully matching probe between subjects. However, the strikingly different amplitude patterns of electrophysiological components elicited during the probes of the left and right hemifield tracking did not translate into differences in performance within the individual subjects.

Recent findings highlight the existence of an object-based tracking mechanism in maintaining the relevant set of targets during a visual tracking task (Merkel et al., 2015, 2017, 2022). Predicated on the polygon idea proposed by Yantis (Yantis, 1992), such a mechanism would build and maintain an abstract, morphing object-based representation of relevant (target) items throughout the tracking phase. A visual probe matching exactly this abstract configuration in a bilateral tracking task was shown to elicit an exclusive modulation of an electrophysiological component—the N170. The sources of this effect originate from the ventral occipital cortical areas (Merkel et al., 2014, 2022). At the same time, the location information for the individual relevant (target) items is maintained during tracking as well, which is indicated by a later component—the N270. Its amplitude modulates with the degree of congruity between probed and tracked items (Merkel et al., 2014, 2022). The current data show a functional and representational dissociation of these two components suggesting the coexistence of two mechanistically different but parallel tracking mechanisms. The N170, indicating an object-based representation of the targets during the probe, was observed when the task was performed within the left hemifield. On the other hand, a modulation of the N270, indicating the maintenance of location-based information, was present during the presentation of the probe when the task was performed within the right hemifield. In the present EEG recordings, these two mechanisms appear to be segregated depending on the spatial location of the tracking task. MOT in the left visual field is performed in an object-based manner while the same task in the right visual field in a location-based manner.

The difference of the sustained CDA modulations following motion onset during the tracking task within the left hemifield and the right hemifield supports this interpretation. The absolute CDA amplitude does correlate with information load during the delay of a working memory task (Vogel and Machizawa, 2004; McCollough et al., 2007) and general bilateral tracking tasks (Drew et al., 2009, 2013). Dynamically changing representations during tracking modulate the CDA hereby “online” (Drew et al., 2012). Compared with the CDA described in visual working memory tasks, the MOT-CDA seems to exhibit a general, load-independent, negative offset which has been argued to be present due to spatial motion (Drew et al., 2011). Such an interpretation would be consistent with the present large negative deflection of the CDA during tracking solely based on item-based spatial information but not abstract object-based information. The currently observed smaller CDA amplitude during left versus right hemifield tracking is furthermore in line with the notion of grouping of the relevant information into an abstract object. Previous work has argued for the CDA to indicate the amount of integrated objects to be maintained (Luria and Vogel, 2011, 2014) and that motion as a feature can facilitate grouping during the retention period (Balaban and Luria, 2015, 2017). Earlier work from our lab dovetails with the currently observed pattern of CDA amplitude modulations for location-based and object-based tracking. In a bilateral tracking task, we observed a sustained γ synchronization during the tracking phase only in subjects employing a location-based tracking strategy, while subjects performing object-based tracking exhibited no such modulation of γ oscillations during the motion phase of the tracking task (Merkel et al., 2022). In general, CDA amplitudes as well as γ oscillations during retention periods were suggested to correlate with information load during the retention period in working memory (Jokisch and Jensen, 2007; Honkanen et al., 2015) and object tracking tasks (Rouhinen et al., 2013).

Hence, maintaining all three individual items during right hemifield tracking indicated by a larger sustained CDA relates to a parametric N270 modulation during the subsequent probe, while during the left hemifield tracking, maintaining the relevant items as one integrated object determines an early match negativity toward the abstract target entity during the probe.

The discussion of hemifield independence in MOT to date revolves around processes with mechanistic restrictions that are equally applicable to both hemifields. In this context, adding relevant items to one of the hemifields during the tracking task adds costs to the contralateral hemisphere in the form of either increased local spatial interference (Alvarez and Cavanagh, 2005; Franconeri et al., 2008; Liu et al., 2009; Stormer et al., 2014), reduced hemifield-specific attentional (multifocal or unifocal) resources (Alvarez and Franconeri, 2007; Howe et al., 2010; Chen et al., 2013; Holcombe and Chen, 2013), or increase in ipsilateral parietal disinhibition (Battelli et al., 2009). The core principle of the bilateral tracking advantage so far is rooted in the idea of a parallel and equally applicable tracking mechanism for the left and right hemifield. Based on the current findings, we propose that the bilateral field advantage during object tracking might arise due to independent modes of representation operating in parallel but with a different functional bias for each hemifield. This bias may be facilitated by hemispheric specialization in representing object-based and location-based visual information.

We observe a hemispheric dissociation of the site of unilateral probe responses with the N170 component mostly originating within the left hemisphere and the N270 amplitude pattern being enhanced within the right hemisphere.

A general perceptual bias for processing location-based and object-based visual information within the right and left hemispheres, respectively, was reported in earlier studies (Marsolek, 1995; Marsolek et al., 1996; Burgund and Marsolek, 1997, 2000). Differential activation patterns within the left and right hemisphere in the processing of abstract and concrete visual information were also observed (McMenamin et al., 2015). Similarly, spatial relations are judged more categorically for stimuli presented to the left hemisphere, while their concrete distance is processed more efficiently when presented to the right hemisphere (Hellige et al., 1989; Kosslyn et al., 1989).

Attentional control mechanisms also exhibit similar differences in hemispherical biases related to object-based and location-based processing. Shifting attention between objects does add additional strain in patients with left parietal lesions but not with right parietal lesion (Egly et al., 1994). Likewise, object-based cueing involves preferentially the cortical attention network of the left hemisphere (Vandenberghe et al., 1997; Arrington et al., 2000; Wilson et al., 2005), while spatial shifts tend to engage the right hemisphere (Fink, 1997; Pollmann and Morrillo, 2003). In a previous functional imaging study, even tracking multiple objects bilaterally seems to show a slight right parietal bias for load-dependent activation over task-dependent activation (Culham et al., 2001; Fig. 3). A study from our group found that patients with left hemisphere lesions showed an N270 response, while it was absent in patients with right hemisphere lesions in a central MOT task. This finding also argues for a functional segregation of representational processes across hemispheres where location-based MOT is dependent on an intact right hemisphere (Lesch et al., 2020).

Importantly, these studies by no means demonstrate a complete functional dissociation between object-based and location-based visual processing, but rather a representational bias for the same information within both hemispheres. Likewise, we observe different amplitude patterns of the N170- and N270-evoked components while tracking within the left or right hemifield across both ipsi- and contralateral, posterior electrodes.

We assume that the extensive interhemispheric communication between visual areas (Dougherty et al., 2005; Shen et al., 2015; Roland et al., 2017) in the current case eventually leads to the very same guided behavioral patterns toward the probe conditions irrespective of the hemifield in which the tracking task occurs. The intriguing situation emerges in which different CDA amplitudes during tracking together with different N170 and N270 amplitude patterns toward probe stimuli within the left or right hemifield do no elicit a different behavioral outcome. Error rates for tracking left and right are almost identical within the subjects. A dissociation of N1 patterns and their associated behavior has been observed in a bilateral tracking task before with different N1 amplitude modulation patterns under different load conditions that nevertheless lead to the same response patterns (Doran and Hoffman, 2010 - Exp. 2/3). Interhemispheric integration of visual processing at later stages of processing would ensure that the differences in representational modes of targets in a hemifield-specific tracking task are compensated for.

In contrast, subjects with improved behavioral responses toward probes that fully match the set of target items (full match) exhibit enhanced N170 response amplitude toward fully matching probes as well. This is consistent with previous descriptions of the N170 during bilateral tracking (Merkel et al., 2014, 2022).

The current results indicate different representational processes for MOT within left and right visual hemifields. These segregated location- and object-based processes are then integrated to underlie a mostly uniform behavioral pattern independent of the spatial location where the task was performed. These findings suggest that the bilateral field advantage during tracking does not necessarily implicate two continuous but separate resources in each hemisphere but rather two different modes of neural representation.

## Data Sharing

All data the analyses and interpretation are based on will be made available online via OSF repository: https://osf.io/z63bd/?view_only=3c93ee1dec2048889be49a6dce541453.

## Synthesis

Reviewing Editor: Niko Busch, Westfalische Wilhelms-Universitat Munster Decisions are customarily a result of the Reviewing Editor and the peer reviewers coming together and discussing their recommendations until a consensus is reached. When revisions are invited, a fact-based synthesis statement explaining their decision and outlining what is needed to prepare a revision will be listed below. The following reviewer(s) agreed to reveal their identity: Roberto Dell'Acqua. Note: If this manuscript was transferred from JNeurosci and a decision was made to accept the manuscript without peer review, a brief statement to this effect will instead be what is listed below.

# Synthesis

The revised manuscript has been reviewed again by two experts on attention and multiple object racking. Both reviews appreciate the substantial revisions and improvements made to the manuscript.

Reviewer 1, who had already reviewed the manuscript previously, was mostly satisfied with the revisions. They recommend a few more important refinements of the statistical analysis, especially regarding the interaction effects and null results.

Reviewer 2 had not reviewed the manuscript in the previous round, but confirmed that you satisfactorily addressed the previous reviewers' comments. However, they raise concerns that strike me as very sensible.

This paradigm includes two relevant intervals.

1: the 4 second long motion interval during which tracking happens

2: the probe interval, during which a comparison happens between tracked objects and the probe objects.

Trials differ by which hemifield was attended, which determines both the hemifield for tracking and the hemifield for comparison. Because the main ERP effect is time-locked to the probe, it is unclear whether this effect reflects a hemifield effect on tracking or comparison. Thus, the results are ambiguous as to whether the hemifields are processed differently during tracking, at comparison, or both.

I strongly recommend acknowledging this ambiguity in the Discussion and to clarify and justify, when discussing the results, whether your conclusions pertain to tracking itself (processes during the tracking interval) or to probe related processes (comparison of previously tracked information).

# Reviewer 1

I have reviewed the previous version of the manuscript, and I found the results in general convincing, and certainly important. There were significant improvements made in this revised version. The CDA analyses especially strengthen the overall story. I still have a few comments:

1) The omnibus ANOVA testing N170 amplitude modulation did not show a significant interaction between match condition and hemifield. In two subsequent one-way ANOVAs it was determined that the effect of match was only significant in the left hemifield. I'm not sure this pattern of results can be described as 'highly dissimilar' between hemifields in light of the non-significant interaction. Interactions of this shape tend to require very high sample sizes for acceptable power, maybe directly comparing M3-M1 effect between the two hemifields in an exploratory way could strengthen the claim. Providing equivalence testing or Bayes factors to quantify absence of the effect would also help. This also applies to the mirroring N270 results.

2) The results of the 2-way ANOVA for high performance and low performance groups do show a significant interaction, which makes the interpretation easier. However, it would help if the test was also described in the manuscript.

3) In the discussion (p.15) it is suggested that multiple object tracking in the right visual field is performed in a "serial, location-based manner". Is there anything in the results that suggests serial tracking, or is it based on previous studies?

# Reviewer 2

I have reviewed the previous version of the manuscript, and I found the results in general convincing, and certainly important. There were significant improvements made in this revised version. The CDA analyses especially strengthen the overall story. I still have a few comments:

1) The omnibus ANOVA testing N170 amplitude modulation did not show a significant interaction between match condition and hemifield. In two subsequent one-way ANOVAs it was determined that the effect of match was only significant in the left hemifield. I'm not sure this pattern of results can be described as 'highly dissimilar' between hemifields in light of the non-significant interaction. Interactions of this shape tend to require very high sample sizes for acceptable power, maybe directly comparing M3-M1 effect between the two hemifields in an exploratory way could strengthen the claim. Providing equivalence testing or Bayes factors to quantify absence of the effect would also help. This also applies to the mirroring N270 results.

2) The results of the 2-way ANOVA for high performance and low performance groups do show a significant interaction, which makes the interpretation easier. However, it would help if the test was also described in the manuscript.

3) In the discussion (p.15) it is suggested that multiple object tracking in the right visual field is performed in a "serial, location-based manner". Is there anything in the results that suggests serial tracking, or is it based on previous studies?

## Author Response

Reviewer #1:

I have reviewed the previous version of the manuscript, and I found the results in general convincing, and certainly important. There were significant improvements made in this revised version. The CDA analyses especially strengthen the overall story. I still have a few comments:

We thank the reviewer for the assessment and are looking forward to clarify any remaining issues.

1) The omnibus ANOVA testing N170 amplitude modulation did not show a significant interaction between match condition and hemifield. In two subsequent one-way ANOVAs it was determined that the effect of match was only significant in the left hemifield. I'm not sure this pattern of results can be described as 'highly dissimilar' between hemifields in light of the non-significant interaction. Interactions of this shape tend to require very high sample sizes for acceptable power, maybe directly comparing M3-M1 effect between the two hemifields in an exploratory way could strengthen the claim.

The reviewer is absolutely right in stating that the lack of a significant interaction between the match conditions with the hemifield for both time frames in the probe response does pose a statistical problem. Post-hoc t-testing to carve out the interaction effect is usually considered 'inelegant'. Looking at the interaction including only two of the match conditions (M2/M3) does confirm the interaction visually, however the conservative significance test does not reach the desired criterion in both cases (N170 and N270). N170: F(1,23) = 1.432, p = 0.234, e = 0.061; N270: F(1,23) = 1.990, p = 0.172, e = 0.080. However, the suggested equivalence test does confirm the observed effect.

Providing equivalence testing or Bayes factors to quantify absence of the effect would also help. This also applies to the mirroring N270 results.

We performed equivalence tests, suggested by the reviewer, on the 2x2-interactions between M3-M2 responses for left and right hemifield tracking. Effect-size bounds were defined as the smallest effect size of interest (SESOI) after Lakens (2014), for which the original test is able to reach significance (a = 0.05). For the interaction of the N170-match responses between left and right hemifield the effect is not significantly different from the upper bound (d = 0.422) with t(23) = -0.845, p = 0.203 while it is different from the lower bound (d = -0.422) with t(23) = 3.29, p = 0.002, suggesting the interaction to potentially reach the size of the upper bound of the SESOI. The N270 interaction effect showed non-equivalency as well, being significantly smaller than the upper bound (t(23) = -3.48, p = 0.001) while at the same time not being larger than the lower bound (t(23) = 0.657, p = 0.259). The non-equivalency is reached for both tests (N170/N270) due to non-significant difference with the upper and lower bound respectively, confirming the visual impression of the N170-effect present in the left hemifield tracking and the N270-effect in right hemifield tracking.

As a comparison, we tested the interactions between N170(M3-M2)-left/N270(M3-M2)--right and N170(M3-M2)-right/N270(M3-M2)-left for equivalency as well (visually both interactions are non-existent). Both tests do not show significance and equivalence with the interaction effects being significantly smaller than the upper bounds and significantly greater than the lower bounds of the SESOI: N170(M3-M2)-left/N270(M3-M2)-right: ub - t(23) = -2.30, p = 0.015, lb - t(23) = 1.84, p = 0.040. N170(M3-M2)-right/N270(M3-M2)-left: ub - t(23) = -2.19 p = 0.019, lb - t(23) = 1.95, p = 0.032.

The equivalence-tests for the N170- and N270-interactions were added to the results in the revised manuscript.

2) The results of the 2-way ANOVA for high performance and low performance groups do show a significant interaction, which makes the interpretation easier. However, it would help if the test was also described in the manuscript.

Thank you for bringing up this point with regard to the behavioral data. We now added the interaction effect between group and match-condition to the results. (F(3,66) = 17.021, p<0.001, e = 0.436) 3) In the discussion (p.15) it is suggested that multiple object tracking in the right visual field is performed in a "serial, location-based manner". Is there anything in the results that suggests serial tracking, or is it based on previous studies? Thank you for this comment. The term 'serial' in this context is meant to include any form of continuous resource model (which can include serial models but not exclude other models). We realize, that the wording infers a potentially temporal process here, which was not at all intended. We deleted the word in the sentence to avoid confusing the reader.

Statistical results of ANOVAs include F and p values, followed by a parameter "e". What does this refer to - is this (partial) Eta square? Please clarify.

Effect sizes for the significant tests refer to (partial) eta square. The formulation is used in the revised manuscript.

Reviewer #2:

I had the opportunity to carefully examine paper and rebuttal, and I must say that as far as the previous reviewers' comments are concerned, the authors did a satisfactory job in changing their ms accordingly.

Given however I have also been asked to lay out my comments, here they are -- I am not sure what would be the impact of a study exploring and making inferences about a particular mental process (tracking) in which the empirical scaffolding is surrected by results mostly obtained after tracking is done, i.e., at the time of probe monitoring.

Please note, that the strategy of using a visual probe to assessing the spatial/non-spatial distribution of attentional resources is widely established. For using that technique in a tracking task it is important to note, that this probe appears precisely at end of the tracking phase, i.e. at a timepoint in which the tracked targets are fully attended. The early electrophysiological responses observed (N170/N270) within occipito-parietal areas are known to reflect attentional modulations (spatial/object-based).

We previously used passive probes during the tracking period as well (Merkel, 2017) to delineate the spread of spatial enhancement relative to the abstract object. For the current research question however we were interested in the general attentional setting in which the targets were processed (location-based vs. object-based) for which the N170 and N270 components are a good indicator (Merkel, 2014, 2022).

I noticed a previous reviewer requested to report CDA activity during the tracking phase of the MOT task, and I must say those results are not informative. Had the authors followd the previous reviewer's advice to discuss their results with reference to Drew and Vogel's CDA results during MOT, they would have incurred a particularly clear demonstration these authors provided that tracking is done in strict collaboration between the two hemisphere, as I commented at length in one of my papers (which I am NOT encouraging the authors to cite...).

Chen, Y., Brigadoi, S., Schiano Lomoriello, A., Jolicœur, P., Simal, A., Fu, S., Baro, V., & Dell'Acqua, R. (2022). A bilateral SPCN is elicited by to‐be‐memorized visual stimuli displayed along the vertical midline. Psychophysiology, 59(8), e14045. https://doi.org/10.1111/psyp.14045 My discussion was focused on the fact it is actually quite risky to assume unilateral processing by either hemisphere because stimuli fall in the contralateral visual field, as the extension of the receptive fields of visual neurons populating high-level portions of the posterior cortex (IPS in particular) is extremely plastic and task-driven. My hunch, after inspecting the CDA results, is that the present CDA functions at best suggest that the RH keeps track of the moving objects for longer whereas the LH perhaps just hands off the load to the RH whenever possible, which is compatible with know asymmetries in attention allocation that are brought to the fore by lesions in posterior high-level processing areas.

We added an extensive discussion about the role of the CDA in the current task in response to the reviewers in the first round of reviews (including the works of Drew/Vogel). We will now expand on that, to address the remaining issues raised.

We believe that our results are highly in line with the MOT-work done by Drew and Vogel in multiple respects. Their task of unilateral tracking (highly similar to the present task) consistently elicited a CDA-difference-waveform that scaled with load. Since they exclusively observed the CDA as a difference wave, no particular statements were made about 'collaboration between the two hemispheres' in their notable work (2008, 2011, 2011, 2013, 2014), the CDA was described as a lateralized component. However, as a functional difference between VWM and MOT they did describe a general CDA-offset (2008), that was load independent, possibly due to the spatial motion unique to the tracking task. This interpretation would be perfectly in line with our data, with a larger CDA amplitude for maintaining spatial information, while object-based representations neither modulate load nor the spatial-tracking specific offset.

Furthermore, in their 2011-jocn work, Drew/Vogel actually made an early prediction about the exact result we describe here in their discussion: "Consider the perceptual grouping effects...perceptual grouping enables participants to effectively lower the number of items that are being tracked...If so, we would expect to observe reduced CDA amplitude".

Lastly the effect of 'hand-off' between hemispheres: it is a potential mechanism in which item representations are shared between hemispheres. This process occurs even before targets cross the meridian and the mentioned overlap described by the reviewer does indeed exist. However, in the current setup it highly unlikely reflects a handoff. In their 2014-paper Drew/Vogel describe specific prerequisites for such a hand-off mechanism, indicated by a CDA-reversal, to occur:

1.: Target items do actually need to traverse the vertical meridian.

2.: There needs to be an expectation/prediction for the item to cross the meridian 3.: The pick-up of a target by the ipsilateral hemisphere does occur not earlier than 1.2{degree sign} before the meridian.

It is important to point out, that none of those conditions are met here. Subjects track unilaterally (no item ever crosses the vertical meridian) within an aperture that is presented peripherally. In the current experiment the inner boundaries of the tracking apertures are positioned 2{degree sign} from the meridian. Thus, according to the 'hand-off' literature, effects to that nature (especially since all targets would have to be covered by the ipsilateral hemisphere throughout the entire tracking phase) cannot explain the results of the current experiment. The paper mentioned by the reviewer in raising the issue of large receptive fields in IPS extending into the contralateral field (Chen, 2022) does discuss an upper boundary of this extension of 2{degree sign}, thus also not indicating that such a mechanism could account for the current data.

## References

[B1] Alvarez GA, Cavanagh P (2005) Independent resources for attentional tracking in the left and right visual hemifields. Psychol Sci 16:637–643. 10.1111/j.1467-9280.2005.01587.x 16102067

[B2] Alvarez GA, Franconeri SL (2007) How many objects can you track? Evidence for a resource-limited attentive tracking mechanism. J Vis 7:14. 10.1167/7.13.14 17997642

[B3] Arrington CM, Carr TH, Mayer AR, Rao SM (2000) Neural mechanisms of visual attention: object-based selection of a region in space. J Cogn Neurosci 12(supplement 2):106–117. 10.1162/089892900563975 11506651

[B4] Balaban H, Luria R (2015) The number of objects determines visual working memory capacity allocation for complex items. NeuroImage 119:54–62. 10.1016/j.neuroimage.2015.06.051 26119024

[B5] Balaban H, Luria R (2017) Neural and behavioral evidence for an online resetting process in visual working memory. J Neurosci 37:1225–1239. 10.1523/JNEUROSCI.2789-16.2016 28011745 PMC6596864

[B6] Balaban H, Luria R (2019) *Using the contralateral delay activity to study online processing of items still within view*. New York: Humana Press.

[B7] Battelli L, Alvarez GA, Carlson T, Pascual-Leone A (2009) The role of the parietal lobe in visual extinction studied with transcranial magnetic stimulation. J Cogn Neurosci 21:1946–1955. 10.1162/jocn.2008.21149 18855545 PMC3366148

[B8] Berg P, Scherg M (1991) Dipole models of eye movements and blinks. Electroencephalogr Clin Neurophysiol 79:36–44. 10.1016/0013-4694(91)90154-V 1713550

[B9] Burgund ED, Marsolek CJ (1997) Letter-case-specific priming in the right cerebral hemisphere with a form-specific perceptual identification task. Brain Cogn 35:239–258. 10.1006/brcg.1997.0940 9356164

[B10] Burgund ED, Marsolek CJ (2000) Viewpoint-invariant and viewpoint-dependent object recognition in dissociable neural subsystems. Psychon Bull Rev 7:480–489. 10.3758/BF03214360 11082854

[B11] Chen W-Y, Howe PD, Holcombe AO (2013) Resource demands of object tracking and differential allocation of the resource. Atten Percept Psychophys 75:710–725. 10.3758/s13414-013-0425-1 23359355

[B12] Culham JC, Cavanagh P, Kanwisher NG (2001) Attention response functions: characterizing brain areas using fMRI activation during parametric variations of attentional load. Neuron 32:737–745. 10.1016/s0896-6273(01)00499-8 11719212

[B13] Dimigen O, Valsecchi M, Sommer W, Kliegl R (2009) Human microsaccade-related visual brain responses. J Neurosci 29:12321–12331. 10.1523/JNEUROSCI.0911-09.2009 19793991 PMC6666125

[B14] Doran MM, Hoffman JE (2010) The role of visual attention in multiple object tracking: evidence from ERPs. Atten Percept Psychophys 72:33–52. 10.3758/APP.72.1.33 20802834 PMC2927139

[B15] Dougherty RF, Ben-Shachar M, Bammer R, Brewer AA, Wandell BA (2005) Functional organization of human occipital-callosal fiber tracts. Proc Natl Acad Sci U S A 102:7350–7355. 10.1073/pnas.0500003102 15883384 PMC1129102

[B16] Drew T, Horowitz TS, Vogel EK (2013) Swapping or dropping? Electrophysiological measures of difficulty during multiple object tracking. Cognition 126:213–223. 10.1016/j.cognition.2012.10.003 23141025 PMC3529852

[B17] Drew T, Horowitz TS, Wolfe JM, Vogel EK (2011) Delineating the neural signatures of tracking spatial position and working memory during attentive tracking. J Neurosci 31:659–668. 10.1523/JNEUROSCI.1339-10.2011 21228175 PMC4486118

[B18] Drew T, Horowitz TS, Wolfe JM, Vogel EK (2012) Neural measures of dynamic changes in attentive tracking load. J Cogn Neurosci 24:440–450. 10.1162/jocn_a_00107 21812558

[B19] Drew T, McCollough AW, Horowitz TS, Vogel EK (2009) Attentional enhancement during multiple-object tracking. Psychon Bull Rev 16:411–417. 10.3758/PBR.16.2.411 19293115 PMC2906218

[B20] Egly R, Driver J, Rafal RD (1994) Shifting visual attention between objects and locations: evidence from normal and parietal lesion subjects. J Exp Psychol Gen 123:161–177. 10.1037/0096-3445.123.2.161 8014611

[B21] Fink G (1997) Space-based and object-based visual attention: shared and specific neural domains. Brain 120:2013–2028. 10.1093/brain/120.11.2013 9397018

[B22] Franconeri SL, Jonathan SV, Scimeca JM (2010) Tracking multiple objects is limited only by object spacing, not by speed, time, or capacity. Psychol Sci 21:920–925. 10.1177/0956797610373935 20534781

[B23] Franconeri SL, Lin JY, Enns JT, Pylyshyn ZW, Fisher B (2008) Evidence against a speed limit in multiple-object tracking. Psychon Bull Rev 15:802–808. 10.3758/PBR.15.4.802 18792507

[B24] Hellige JB, Taylor AK, Eng TL (1989) Interhemispheric interaction when both hemispheres have access to the same stimulus information. J Exp Psychol Hum Percept Perform 15:711–722. 10.1037//0096-1523.15.4.711 2531206

[B25] Holcombe AO, Chen W-Y (2012) Exhausting attentional tracking resources with a single fast-moving object. Cognition 123:218–228. 10.1016/j.cognition.2011.10.003 22055340

[B26] Holcombe AO, Chen W-Y (2013) Splitting attention reduces temporal resolution from 7 Hz for tracking one object to <3 Hz when tracking three. J Vis 13:12–12. 10.1167/13.1.12 23302215

[B27] Holcombe AO, Chen W-Y, Howe PDL (2014) Object tracking: absence of long-range spatial interference supports resource theories. J Vis 14:1–1. 10.1167/14.6.1 25086084

[B28] Honkanen R, Rouhinen S, Wang SH, Palva JM, Palva S (2015) Gamma oscillations underlie the maintenance of feature-specific information and the contents of visual working memory. Cereb Cortex 25:3788–3801. 10.1093/cercor/bhu263 25405942

[B29] Horowitz TS, Cohen MA (2010) Direction information in multiple object tracking is limited by a graded resource. Atten Percept Psychophys 72:1765–1775. 10.3758/APP.72.7.1765 20952776 PMC2957661

[B30] Howe PDL, Cohen MA, Pinto Y, Horowitz TS (2010) Distinguishing between parallel and serial accounts of multiple object tracking. J Vis 10:11–11. 10.1167/10.8.11 PMC2951291 20884586

[B31] Jokisch D, Jensen O (2007) Modulation of gamma and alpha activity during a working memory task engaging the dorsal or ventral stream. J Neurosci 27:3244–3251. 10.1523/JNEUROSCI.5399-06.2007 17376984 PMC6672464

[B32] Keren AS, Yuval-Greenberg S, Deouell LY (2010) Saccadic spike potentials in gamma-band EEG: characterization, detection and suppression. NeuroImage 49:2248–2263. 10.1016/j.neuroimage.2009.10.057 19874901

[B33] Kosslyn SM, Koenig O, Barrett A, Cave CB, Tang J, Gabrieli JDE (1989) Evidence for two types of spatial representations: hemispheric specialization for categorical and coordinate relations. J Exp Psychol Hum Percept Perform 15:723–735. 10.1037/0096-1523.15.4.723 2531207

[B34] Lakens D (2014) Performing high-powered studies efficiently with sequential analyses. Eur J Soc Psychol 44:701–710. 10.1002/ejsp.2023

[B35] Lesch H, Schoenfeld MA, Merkel C (2020) Functional dissociation of multiple-object tracking mechanisms based on hemispheric asymmetries. Restor Neurol Neurosci 38:443. 10.3233/RNN-201048 33325416

[B36] Liu T, Jiang Y, Sun X, He S (2009) Reduction of the crowding effect in spatially adjacent but cortically remote visual stimuli. Curr Biol 19:127–132. 10.1016/j.cub.2008.11.065 19135367 PMC3175242

[B37] Luria R, Vogel EK (2011) Shape and color conjunction stimuli are represented as bound objects in visual working memory. Neuropsychologia 49(6):1632–1639. 10.1016/j.neuropsychologia.2010.11.031 21145333 PMC3095682

[B38] Luria R, Vogel EK (2014) Come together, right now: dynamic overwriting of an object’s history through common fate. J Cogn Neurosci 26:1819–1828. 10.1162/jocn_a_00584 24564468 PMC4486205

[B39] Marsolek CJ (1995) Abstract visual-form representations in the left cerebral hemisphere. J Exp Psychol Hum Percept Perform 21:375–386. 10.1037//0096-1523.21.2.375 7714478

[B40] Marsolek CJ, Schacter DL, Nicholas CD (1996) Form-specific visual priming for new associations in the right cerebral hemisphere. Mem Cognit 24:539–556. 10.3758/BF03201082 8870526

[B41] McCollough AW, Machizawa MG, Vogel EK (2007) Electrophysiological measures of maintaining representations in visual working memory. Cortex 43:77–94. 10.1016/S0010-9452(08)70447-7 17334209

[B42] McMenamin BW, Deason RG, Steele VR, Koutstaal W, Marsolek CJ (2015) Separability of abstract-category and specific-exemplar visual object subsystems: evidence from fMRI pattern analysis. Brain Cogn 93:54–63. 10.1016/j.bandc.2014.11.007 25528436 PMC4281302

[B43] Merkel C, Hopf J-M, Heinze H-J, Schoenfeld MA (2015) Neural correlates of multiple object tracking strategies. NeuroImage 118:63–73. 10.1016/j.neuroimage.2015.06.005 26054872

[B44] Merkel C, Hopf J-M, Schoenfeld MA (2017) Spatio-temporal dynamics of attentional selection stages during multiple object tracking. NeuroImage 146:484–491. 10.1016/j.neuroimage.2016.10.046 27810524

[B45] Merkel C, Hopf J-M, Schoenfeld MA (2020) *Spatial learning and attention guidance* (Pollmann S, ed), pp 157–176. New York: Springer US.

[B46] Merkel C, Hopf J-M, Schoenfeld MA (2022) Electrophysiological hallmarks of location-based and object-based visual multiple objects tracking. Eur J Neurosci 55:1200. 10.1111/ejn.15605 35075713

[B47] Merkel C, Stoppel CM, Hillyard SA, Heinze H-J, Hopf J-M, Schoenfeld MA (2014) Spatio-temporal patterns of brain activity distinguish strategies of multiple-object tracking. J Cogn Neurosci 26:28–40. 10.1162/jocn_a_00455 23915053

[B48] Picton TW, van Roon P, Armilio ML, Berg P, Ille N, Scherg M (2000) The correction of ocular artifacts: a topographic perspective. Clin Neurophysiol 111:53–65. 10.1016/S1388-2457(99)00227-8 10656511

[B49] Pollmann S, Morrillo M (2003) Left and right occipital cortices differ in their response to spatial cueing. NeuroImage 18:273–283. 10.1016/S1053-8119(02)00039-3 12595182

[B50] Pylyshyn Z (1989) The role of location indexes in spatial perception: a sketch of the FINST spatial-index model. Cognition 32:65–97. 10.1016/0010-0277(89)90014-0 2752706

[B51] Pylyshyn Z (2004) Some puzzling findings in multiple object tracking: I. tracking without keeping track of object identities. Vis. Cogn. 11:801–822. 10.1080/13506280344000518

[B52] Pylyshyn ZW, Storm RW (1988) Tracking multiple independent targets: evidence for a parallel tracking mechanism*. Spat Vis 3:179–197. 10.1163/156856888X00122 3153671

[B53] Roland JL, Snyder AZ, Hacker CD, Mitra A, Shimony JS, Limbrick DD, Raichle ME, Smyth MD, Leuthardt EC (2017) On the role of the corpus callosum in interhemispheric functional connectivity in humans. Proc Natl Acad Sci U S A 114:13278–13283. 10.1073/pnas.1707050114 29183973 PMC5740665

[B54] Rouhinen S, Panula J, Palva JM, Palva S (2013) Load dependence of β and γ oscillations predicts individual capacity of visual attention. J Neurosci 33:19023–19033. 10.1523/JNEUROSCI.1666-13.2013 24285906 PMC6618707

[B55] Scholl BJ, Pylyshyn ZW, Feldman J (2001) What is a visual object? Evidence from target merging in multiple object tracking. Cognition 80:159–177. 10.1016/S0010-0277(00)00157-8 11245843

[B56] Shen K, et al. (2015) Stable long-range interhemispheric coordination is supported by direct anatomical projections. Proc Natl Acad Sci U S A 112:6473–6478. 10.1073/pnas.1503436112 25941372 PMC4443345

[B57] Shim WM, Alvarez GA, Jiang YV (2008) Spatial separation between targets constrains maintenance of attention on multiple objects. Psychon Bull Rev 15:390–397. 10.3758/PBR.15.2.390 18488657 PMC2621007

[B58] Stormer VS, Alvarez GA, Cavanagh P (2014) Within-hemifield competition in early visual areas limits the ability to track multiple objects with attention. J Neurosci 34:11526–11533. 10.1523/JNEUROSCI.0980-14.2014 25164651 PMC4145167

[B59] Strong RW, Alvarez GA (2020) Hemifield-specific control of spatial attention and working memory: evidence from hemifield crossover costs. J Vis 20:24–24. 10.1167/jov.20.8.24 PMC7453044 32841317

[B60] Vandenberghe R, Duncan J, Dupont P, Ward R, Poline J-B, Bormans G, Michiels J, Mortelmans L, Orban GA (1997) Attention to one or two features in left or right visual field: a positron emission tomography study. J Neurosci 17:3739–3750. 10.1523/JNEUROSCI.17-10-03739.1997 9133394 PMC6573671

[B61] Vogel EK, Machizawa MG (2004) Neural activity predicts individual differences in visual working memory capacity. Nature 428:748–751. 10.1038/nature02447 15085132

[B62] Wilson KD, Woldorff MG, Mangun GR (2005) Control networks and hemispheric asymmetries in parietal cortex during attentional orienting in different spatial reference frames. NeuroImage 25:668–683. 10.1016/j.neuroimage.2004.07.075 15808968

[B63] Yantis S (1992) Multielement visual tracking: attention and perceptual organization. Cogn Psychol 24:295–340. 10.1016/0010-0285(92)90010-Y 1516359

